# Treatment outcome in patients with stage III breast cancer treated with neoadjuvant chemotherapy

**DOI:** 10.3892/etm.2013.1289

**Published:** 2013-09-06

**Authors:** RYUJI TAKAHASHI, UHI TOH, NOBUTAKA IWAKUMA, MAI MISHIMA, TERUHIKO FUJII, MIKI TAKENAKA, KEIKO KOURA, NAOKO SEKI, AKIHIKO KAWAHARA, MASHAYOSHI KAGE, ETSUYO OGO, KAZUO SHIROUZU

**Affiliations:** 1 Department of Surgery, Kurume University School of Medicine, Kurume, Fukuoka 830-0011, Japan; 2 Research Center for Innovative Cancer Therapy, Kurume University School of Medicine, Kurume, Fukuoka 830-0011, Japan; 3 Departments of Pathology, Kurume University School of Medicine, Kurume, Fukuoka 830-0011, Japan; 4 Radiology, Kurume University School of Medicine, Kurume, Fukuoka 830-0011, Japan

**Keywords:** treatment outcome, prognostic indicator, neoadjuvant chemotherapy, stage III breast cancer

## Abstract

Despite the good responses of patients (pts) with stage III breast cancer to neoadjuvant chemotherapy (NAC), most eventually relapse and have a poor prognosis. We investigated the prognostic indicators in pts with stage III breast cancer treated with NAC, using epirubicin and/or docetaxel. A total of 22 women with stage III breast cancer underwent NAC between January 2005 and May 2011. The regimens of NAC comprised ED (epirubicin 60 mg/m^2^ and docetaxel 60 mg/m^2^) in 10 cases, FEC (fluorouracil 500 mg/m^2^, epirubicin 75–100 mg/m^2^ and cyclophosphamide 500 mg/m^2^) in 10 cases and EC (epirubicin 60 mg/m^2^ and cyclophosphamide 600 mg/m^2^) in two cases. Following four cycles of each regimen, a further four cycles of D (docetaxel 70 mg/m^2^) were undertaken in nine cases. Subsequent to the completion of NAC and surgery, we assessed the clinicopathological results and performed prognostic analyses. Statistical analyses concerning disease-free survival (DFS) or overall survival (OS) were conducted by a Cox proportional hazard model. The median survival time was 66 months and there were 12 distant metastases and two local recurrences. Multivariate analyses showed the number of metastatic axillary lymph nodes (ALNs) [hazard ratio (HR), 1.079; P=0.023] was correlated with DFS, while the Ki-67 labeling index (HR, 1.109; P=0.042) and the number of meta-static ALNs (HR, 1.087; P=0.023) were correlated with OS. In conclusion, even if pts with stage III breast cancer show good responses to NAC using epirubicin and/or docetaxel, the majority eventually relapse and have a poor prognosis. The Ki-67 labeling index and the number of involved ALNs are suggested as prognostic indicators in stage III breast cancer.

## Introduction

Neoadjuvant chemotherapy (NAC) has become a standard therapy for patients (pts) with locally advanced breast cancer. Despite the initial good responses of pts with stage III breast cancer to NAC, these pts tend to relapse earlier and have worse prognoses than pts with stage I/II breast cancer. According to the databases of the American Cancer Society (1), the five-year overall survival (OS) rates for stage III breast cancer are 67% in stage IIIA and 41–49% in stage IIIB–IIIC. A number of prognostic factors for NAC have been correlated with OS and disease free survival (DFS) in locally advanced breast cancer, such as the triple-negative type, the human epidermal growth factor receptor 2 (HER2)-enriched type (hormone receptor negative/HER2 positive type) (2,3), a pathological complete response (pCR) (4,5) and the number of involved axillary lymph nodes (ALNs) at surgical staging (6,7). However, only a small number of studies have investigated the prognostic indicators that are associated with long-term survival in pts with stage III breast cancer treated with NAC. The aim of this small-scale study was to investigate the prognostic indicators in pts with stage III breast cancer who have been treated with NAC.

## Patients and methods

### Study design and approval

This study was designed as an analysis of retrospective data in a single-facility, the Kurume University School of Medicine (Kurume, Japan). This observational study was approved by the Ethics Committee of Kurume University and all pts provided written informed consent for the treatment and publication of the data.

### Eligibility criteria

Women under the age of 75 years with previously untreated clinical stage III breast cancer, who were diagnosed by mammography, ultrasonography, breast magnetic resonance imaging (MRI), core needle biopsy and positron emission tomography-computed tomography (PET/CT), were eligible for this study. Each pt had a locally advanced breast cancer with ALN involvement. The eligibility criteria also included adequate performance status [Eastern Cooperative Oncology Group (ECOG) performance 0–1], adequate hematology, renal and liver function and an ejection fraction ≥60%, confirmed by ultrasonic cardiography. Pts who had an otherwise adverse medical history, another malignancy, contralateral breast cancer and/or a severe systemic condition were excluded.

### NAC regimens and surgical methods

Three different NAC regimens were used: ED (60 mg/m^2^ epirubicin and 60 mg/m^2^ docetaxel), FEC (500 mg/m^2^ fluorouracil, 75–100 mg/m^2^ epirubicin, and 500 mg/m^2^ cyclophosphamide) and EC (60 mg/m^2^ epirubicin and 600 mg/m^2^ cyclophosphamide). For the pts who underwent EC and most of the pts who underwent FEC, a further four cycles of D (docetaxel 70 mg/m^2^) were then administered. Each chemotherapy regimen was administered every three weeks for four cycles; however, this interval was prolonged by at least one week if the pt did not recover from the adverse effects. Subsequent to the completion of the four cycles of NAC, we evaluated the clinical responses and performed surgery within 2–3 weeks. The surgical methods included Patey’s procedure in three pts, mastectomy in 16 pts and lumpectomy in three pts. All pts underwent level I+II ALN dissection. In addition, the three pts who received Patey’s procedure underwent level III lymph node dissection.

### Adjuvant therapy after surgery

Following surgery, extensional adjuvant chemotherapy was administered to 13/22 pts (59%) who had numerous ALN metastases (≥4 positive nodes) and/or poor pathological responses to NAC. Each regimen of extensional chemotherapy was selected by the clinician. Nine of the 22 pts (41%), who had a positive HER2 status, were treated with adjuvant trastuzumab (initially 8 mg/kg, followed by 6 mg/kg) for 12 months. Subsequent to the completion of adjuvant chemotherapy, whole breast irradiation of 50 Gy was performed for the pts who underwent a lumpectomy, while chest wall and regional lymph node irradiation of 50–60 Gy was performed for the majority of the pts. In addition, postmenopausal pts were treated with aromatase inhibitors for ≥5 years, whereas premenopausal pts were given tamoxifen until menopause, prior to being switched to aromatase inhibitors.

### Assessment of NAC in stage III breat cancer

To compare the efficacy of NAC using epirubicin and/or docetaxel in stage III breast cancer, we investigated 31 pts with stage III breast cancer who were treated with adjuvant chemotherapy between 1996 and 2005.

### Evaluation of chemotherapy responses and toxicities

The clinical response was assessed based on a physical examination, mammography, ultrasonography, MRI and CT according to the Response Evaluation Criteria In Solid Tumors (RECIST) version 1.1 criteria (8). A clinically complete response (cCR) was defined as the disappearance of all known lesions; a clinically partial response (cPR) was defined as a ≥30% reduction in the sum of the longest diameter (LD) of the primary lesion; progressive disease (PD) was defined as a ≥20% increase in the sum of the LD of the primary lesion and stable disease (SD) was defined as neither sufficient shrinkage to qualify for cPR nor sufficient increase to qualify for PD. The efficacy of NAC was examined in the surgical specimens, while the Ki-67 labeling index was examined in the pre-treatment biopsy specimens. The pathological response was assessed based on the histological changes in the invasive area by the Japanese Breast Cancer Society criteria (9). A pCR was defined as no residual invasive cancer in the breast tissue, regardless of the ALN status, while the grade 0 response indicated no cancerous degeneration. A grade 2 response was defined as ≥2/3 cancerous degeneration or a small amount of invasive cancer in the specimen, while a grade 1 response was defined as <2/3 cancerous degeneration in the specimen. The number of involved ALNs was confirmed in the dissected ALN specimen by the pathologist. In addition, toxicities of the NAC were graded by the ECOG common toxicity criteria.

### Statistical analysis

OS and DFS were considered from the onset of NAC and from the day of breast surgery, respectively. The statistical analysis was conducted using JMP version 9.0 statistical software (SAS Institute, Inc., Cary, NC, USA). The correlation analysis was performed to compare two variables, including categorical and continuous variables. Univariate survival analyses to investigate predictive factors for OS and DFS were performed with a Cox proportional hazard model. The significant factors (P<0.05) were entered into a Cox multivariate regression model to analyze the potential simultaneous effects of the predictors of OS and DFS identified by univariate analyses. The survival analysis of the most significant factor was performed with the Kaplan-Meier method, and comparisons between the survival curves were performed with the log-rank test. Pt follow-up was performed in our hospital, from the beginning of chemotherapy either until mortality or the last visit of the pt.

## Results

### Pt characteristics

A total of 22 women with stage III breast cancer underwent NAC between January 2005 and May 2011. The median age was 55 years (range, 33–72 years) and the median follow-up period was 66 months (range, 9.3–90.0 months). The clinicopathological characteristics of the pts are shown in Table I. The observed intrinsic subtypes were as follows: six luminal, nine HER2-positive and seven triple-negative types. The pts’ histological types showed invasive ductal carcinoma in 19 pts, mucinous carcinoma in two pts and invasive lobular carcinoma in one pt. There were no significant differences between the pts who underwent the FEC regimen and those who underwent a non-FEC regimen (ED, EC followed by D).

### Clinical/pathological responses and toxicities

Clinical efficacy of NAC was observed in 64% (cCR, 5%; cPR, 59%) of all pts (Table I). The remaining 36% had SD (27%) and PD (9%). A younger age (P=0.010) and non-FEC regimen (P=0.005) were correlated with poor clinical responses. Pathological efficacy was observed in 36% (pCR, 18%; grade 2, 18%) of all pts (Table I). The remaining 64% showed grade 1 (50%) and grade 0 (14%) responses. There was no significant factor predicting whether the pathological response was likely to be good or poor.

Based on the ECOG common toxicity criteria, the most common toxicities were grade 1 and 2 neutropenia (n=12, Table II). One pt required an admission for grade 4 neutropenia, and six pts were classified as grade 3. The other grade 3 toxicities are shown in Table II. Additional common toxicities were anorexia (n=7) and fever (n=6). The majority of the pts experienced alopecia.

### Prognostic factors associated with OS and DFS

The median survival time was 66 months, and 11/22 pts (50%) succumbed to refractory breast cancer. There were 12 distant metastases (five brain metastases, five lung metastases, one bone metastasis and one subclavian lymph nodes metastasis) and two local recurrences. pCR was not a prognostic factor for the success of NAC in this study. Univariate analyses showed that the triple-negative type, positive status of estrogen receptor and the number of involved ALNs were correlated with DFS, while the triple-negative type, Ki-67 labeling index (%), pathological tumor size (cm) and the number of involved ALNs were correlated with OS (Table III). Multivariate analyses showed that the number of involved ALNs [hazard ratio (HR), 1.079; 95% confidence interval (CI), 1.011–1.155; P= 0.023] was correlated with DFS, while the Ki-67 labeling index (HR, 1.109; 95% CI, 1.004–1.265; P=0.042) and the number of involved ALNs (HR, 1.087; 95% CI, 1.012–1.180; P=0.023) were correlated with OS (Table III).

### Treatment outcome of the pts with confirmed pCR

We assessed the outcome of four pts with observed pCR subsequent to surgery (Table IV). The five-year survival rate of these pts was 75% (3/4 pts) and 2/4 pts (50%) suffered from a relapse of the breast cancer. These two pts relapsed with brain metastasis, having had a short DFS (2.9 and 7.9 months). For the four pts with observed pCR subsequent to surgery, initial staging, intrinsic subtype and Ki-67 labeling index were suggested as prognostic indicators.

### Feasibility of NAC in stage III breast cancer

**Figure 1.**
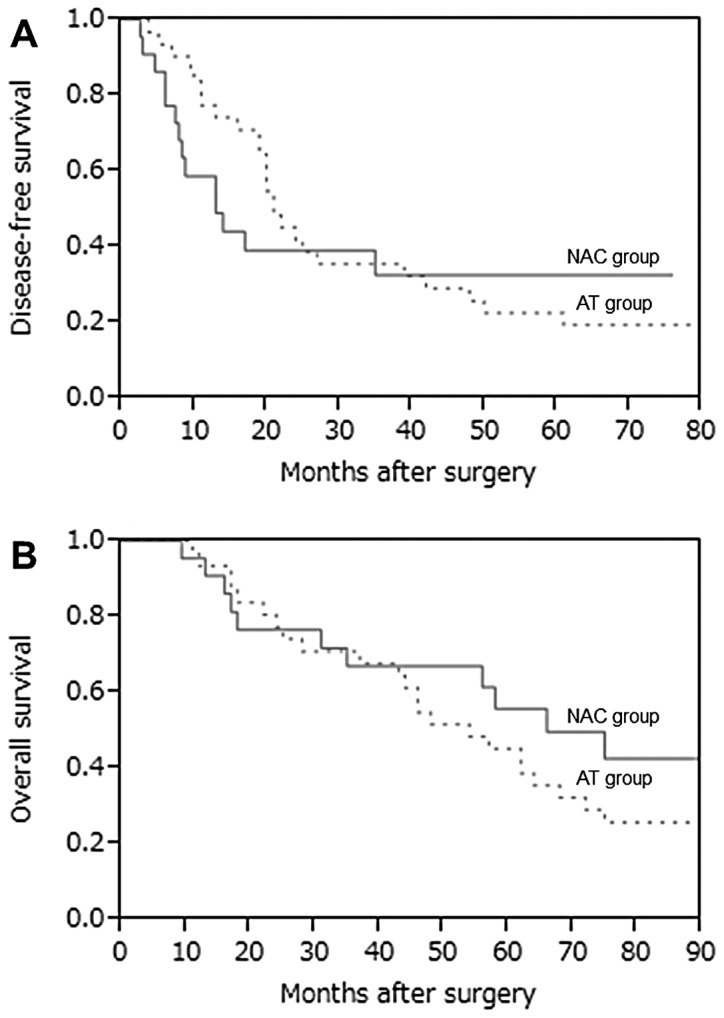
(A) Disease-free (DFS) and (B) overall survival (OS) curves for the neoadjuvant chemotherapy (NAC) and adjuvant therapy (AT) groups. No significant difference was identified between the two groups in the DFS and OS curves (P=0.813 and P=0.328, respectively). The median survival time was 66 months in the NAC group and 54 months in the AT group.

Comparisons of the characteristics for the pts treated with NAC and those treated with adjuvant therapy (AT) are shown in Table V. The pts treated with AT included a higher proportion of stage IIIA disease and a smaller proportion of stage IIIC disease (P=0.040). The majority of the pts treated with AT underwent four cycles of anthracycline- and/or taxane-based regimens using doxorubicin, epirubicin and/or paclitaxel. DFS and OS curves for the NAC and AT groups are shown in Fig. 1. In our hospital, epirubicin and/or docetaxel-based NAC was found not contribute to enhanced survival in stage III breast cancer.

### Prognostic indicators in stage III breast cancer pts treated with NAC

**Figure 2.**
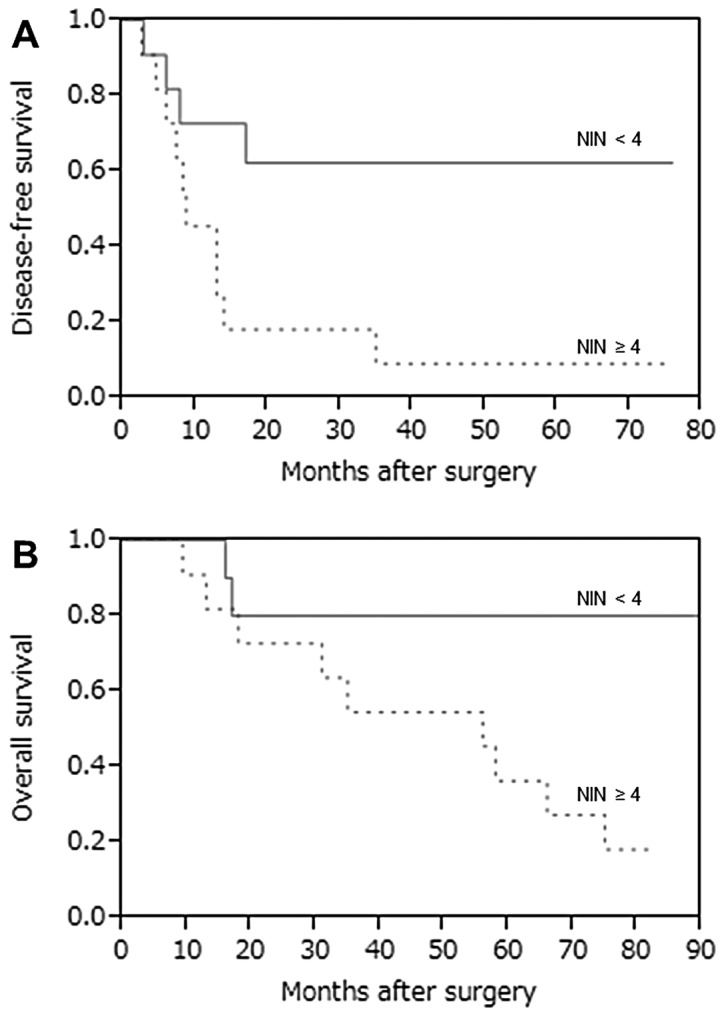
(A) Disease-free (DFS) and (B) overall survival (OS) curves for patients with different numbers of involved lymph nodes (NIN). A high NIN (≥4) was a significant prognostic factor correlated with DFS and OS (P=0.025 and P=0.024, respectively).

**Figure 3.**
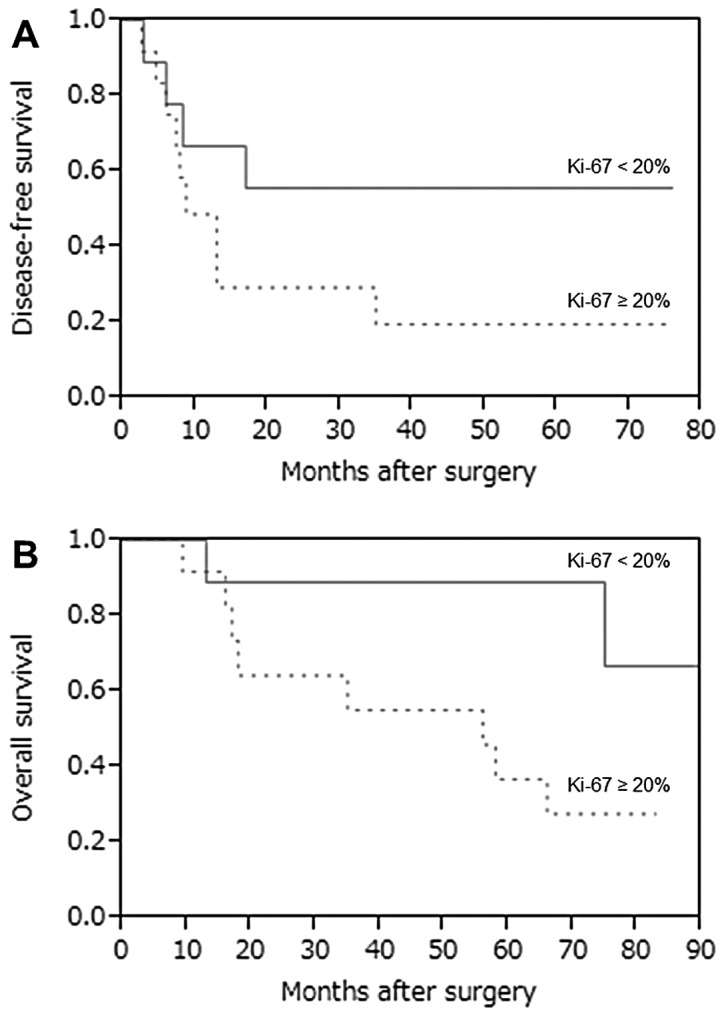
(A) Disease-free (DFS) and (B) overall survival (OS) curves according to the percentage of Ki-67. A high percentage of Ki-67 (≥20%) appears to be as a prognostic factor correlated with OS (P=0.057), while no significant difference between low and high percentages of Ki-67 was observed in the DFS curves (P=0.183).

We compared DFS and OS curves between pts with NIN <4 and pts with NIN ≥4, and a greater number of NIN (≥4) was significantly correlated with poor prognoses. Tausch *et al* (10) observed that an increased number of involved nodes (NIN) and an increased ratio of involved to removed nodes (LNR) were significantly correlated with worse DFS and OS in univariate and multivariate analyses (P<0.001). We compared DFS and OS curves between pts with NIN <4 and pts with NIN ≥4 (Fig. 2). A high number of NINs (≥4) was a significant prognostic indicator correlated with DFS and OS (P=0.025 and P=0.024, respectively). In the current study, the Ki-67 index was indicated to be as an independent prognostic factor for OS (HR, 1.109; 95% CI, 1.004–1.265; P=0.042). However, achieving pCR subsequent to NAC was not associated with the Ki-67 labeling index (P=0.654, Wilcoxon test). Since the median percentage of Ki-67 was 21.3% (range, 7.1–55.2%), we compared the DFS and OS curves with a cut-off value of Ki-67 at 20% (Fig. 3). A high percentage of Ki-67 (≥20%) was suggested as a prognostic indicator correlated with OS (P=0.057), while there was no significant difference between the DFS curves (P=0.183).

## Discussion

According to our five years of follow-up data for NAC in stage III breast cancer, the five-year OS and DFS rates were 50 and 36.4%, respectively. The pCR and breast conserving rates after NAC were 18.2 and 13.6%, respectively. Chávez-MacGregor and González-Angulo (4) suggested that achieving pCR after NAC correlated with improved DFS and OS and that, therefore, the amount of residual disease was a prognostic predictor. Ionta *et al* (11) analyzed 58/74 consecutive pts with stage IIIB breast cancer, who failed to achieve pCR following up to six cycles of a primary cisplatin, epirubicin and vinorelbine regimen. Following a median follow-up of 99 months, the 10-year DFS and OS rates were 37.6 and 50.3%, respectively, which were significantly worse than those in the pCR group (n=16; P=0.003 and P=0.008, respectively). Their results suggested that the number of residual ALNs and being negative for hormone receptors were strong predictors of poor outcomes, while the triple-negative type showed a trend towards early recurrence and mortality. Our results also suggested that the pathological tumor size subsequent to NAC and a triple-negative type were prognostic predictors. However, no significant difference was observed in the multivariate analysis. It was not possible to evaluate these predictors accurately; therefore, larger numbers of pts with stage III breast cancer treated with NAC are required for analysis.

With regard to the validity of NAC in stage III breast cancer, Tanioka *et al* (12) investigated the predictive factors of recurrence in 88 pts achieving pCR following NAC. During a median 46-month follow-up period, there were 12 recurrences, including eight distant metastases. Multivariate analyses showed that ALN metastasis (HR, 13.6; P<0.001) and HER2-positive type (HR, 5.0; P=0.019) were significant predictors of recurrence. In the current study, we observed two pts who experienced relapses of breast cancer into the brain out of the four pts who achieved pCR after NAC (Table IV). Although these two pts had a small number of involved ALNs, they were stage IIIC; one pt had a HER2-positive type and the other had a high percentage of Ki-67 (55.2%). Yuan *et al* (13) revealed that NAC exhibited better recurrence control and DFS and OS rates than adjuvant chemotherapy in stage III breast cancer; however, it did not result in greater survival in stage II disease. By contrast, our results indicated that NAC may have no survival advantages compared with adjuvant chemotherapy in stage III breast cancer pts. Notably, higher frequencies of the triple-negative type and stage IIIB-IIIC breast cancer were shown in the NAC group. However, these results indicate the need to consider more tailored and effective NAC regimens for pts with stage III breast cancer.

Our results suggested that the Ki-67 labeling index and the number of involved ALNs are prognostic predictors in stage III breast cancer. In a recent study, Zhang *et al* (7) investigated axillary nodal staging in stage II/III breast cancer after NAC. The authors observed that the ypN staging adjusted by pCR following NAC may predict differential DFS. We showed that the ypN staging after NAC may be a prognostic indicator among stage III breast cancer pts, although the number of pts was too small to confirm this. A greater number of NIN (≥4) after NAC may also be a predictive factor for recurrence or poor prognosis in our study. Furthermore, we evaluated the Ki-67 in pre-treatment biopsy specimens, due to the fact that tissue degeneration following chemotherapy often makes it difficult to identify Ki-67-positive tumor cells. In a recent review of Ki-67 data (14), Ki-67 was an independent prognostic factor for DFS (HR, 1.05-1.72) in multivariate analyses in seven randomized trials (level of evidence, I-B) and for OS (HR, 1.11–1.83) in univariate analyses in five trials. In addition, a high Ki-67 was observed to be correlated with immediate pCR with neoadjuvant therapy (level of evidence, II-B). I-B and II-B levels of evidence may be defined as follows: The I-B level of evidence applies to instances where a randomized controlled trial (RCT) was not specifically performed to assess the utility of the biomarker. The samples were stored during the study and analyzed when the study had finished, following a protocol. Only one validation study, or several studies with inconsistent results were desirable. For a II-B level of evidence, an RCT was not specifically performed to assess the utility of the biomarker. The samples were stored during the study and analyzed once the study had finished, following a protocol. One or more validation studies with consistent results were desirable. In the present study, the Ki-67 was indicated to be an independent prognostic factor for OS, and a high percentage of Ki-67 (≥20%) was correlated with poor prognosis, although there was no significant difference between the DFS curves. These results indicate that the Ki-67 index in pre-treatment tumor tissues may be used as a prognostic indicator for localized advanced breast cancer pts.

In conclusion, even if pts with stage III breast cancer show good responses to NAC using anthracycline and/or taxanes, most eventually relapse and have a poor prognosis. The Ki-67 labeling index and the number of involved ALNs may be prognostic indicators in stage III breast cancer.

## Figures and Tables

**Table I.** Characteristics of the patients treated with NAC.

Characteristic	n (%)^a^
Median age [years (range)]	55 (33–72)
Menopausal status	
Premenopause	9 (41)
Postmenopause	13 (59)
Histological type	
Invasive ductal carcinoma	19 (86)
Mucinous carcinoma	2 (9)
Invasive lobular carcinoma	1 (5)
Nuclear grade	
I–II	14 (64)
III	8 (36)
Intrinsic subtype	
Luminal	6 (27)
HER2-positive	9 (41)
Triple negative	7 (32)
NAC regimen	
FEC (+D)	10 (45)
ED	10 (45)
EC+D	2 (10)
Initial tumor size (cm)	
≤2	3 (13)
>2, ≤5	9 (41)
>5	10 (46)
Initial axillary nodal status	
N1	6 (27)
N2	11 (50)
N3	5 (23)
Clinical stage	
Stage IIIA	10 (45)
Stage IIIB	7 (32)
Stage IIIC	5 (23)
Surgical method	
Lumpectomy	3 (13)
Mastectomy	16 (73)
Patey’s procedure	3 (13)
Radiotherapy	
Yes	16 (73)
No	6 (27)
Clinical response	
CR/PR	1/13 (5/59)
SD/PD	6/2 (27/9)
Pathological response	
pCR/grade 2	4/4 (18/18)
grade 0/grade 1	3/11 (14/50)

NAC, neoadjuvant chemotherapy; FEC, 75–100 mg/m^2^ epirubicin, 500 mg/m^2^ fluorouracil, 500 mg/m^2^ cyclophosphamide; D, docetaxel 70 mg/m^2^; ED, 60 mg/m^2^ epirubicin, 60 mg/m^2^ docetaxel; EC, 60 mg/m^2^ epirubicin, 600 mg/m^2^ cyclophosphamide; CR, complete resonse; PR, partial response; SD, stable disease; PD, progressive disease; pCR, pathological complete response.

a For age, the range not % is shown.

**Table II.** Adverse events of NAC.

Adverse event	Grade 1–2	Grade 3	Grade 4
Constitutinal symptom			
Fever	5	1	
Malaise	2		
Gastrointestinal			
Anorexia	7		
Nausea	4		
Diarrhea	3		
Oral mucositis	3		
Neurological			
Dysgeusia	3		
Stroke	1		
Blood/bone marrow			
Anemia	3	1	
Neutropenia	12	6	1
Thrombocytopenia		1	
Laboratory			
AST/ALT elevation	2		

NAC, neoadjuvant chemotherapy; AST, aspartate aminotransferase; ALT, alanine aminotransferase.

**Table III.** Uni- and multivariate analyses of the clinicopathological factors associated with overall and disease-free survival.

Factor	Overall survival				Disease-free survival			
	Univariate		Multivariate		Univariate		Multivariate	
	HR	P-value	HR (95% CI)	P-value	HR	P-value	HR (95% CI)	P-value
Menopausal status								
Post-/premenopause	0.400	0.135			0.466	0.161		
NAC regimen								
FEC/non-FEC regimen	1.081	0.903			0.595	0.344		
Initial stage								
IIIB-IIIC/IIIA	0.835	0.769			1.343	0.584		
Nuclear grade								
III/I-II	1.591	0.450			1.412	0.528		
Triple-negative type								
Yes/no	27.99	<0.001	9.905 (0.692–274.6)	0.091	3.329	0.047	1.206 (0.111–7.789)	0.857
Estrogen receptor								
Positive/negative	0.336	0.123			0.167	0.006	0.273 (0.037–1.388)	0.118
HER2 status								
Positive/negative	0.366	0.113			1.310	0.615		
Ki-67 labeling index (%)	1.120	<0.001	1.109 (1.004–1.265)	0.042	1.048	0.057		
Pathological tumor size (cm)	1.605	0.006	1.242 (0.895–1.826)	0.194	1.350	0.057		
Involved ALNs (number)	1.061	0.037	1.087 (1.012–1.180)	0.023	1.068	0.027	1.079 (1.011–1.155)	0.023
Pathological responses								
pCR, grade 2/grade 1, grade 0	0.348	0.139			0.383	0.112		

FEC, 75–100 mg/m^2^ epirubicin, 500 mg/m^2^ fluorouracil, 500 mg/m^2^ cyclophosphamide; HR, hazard ratio; CI, confidence interval; NAC, neoadjuvant chemotherapy; HER2, human epidermal growth factor receptor 2; ALNs, axillary lymph nodes; pCR, pathological complete response.

**Table IV.** Outcomes of the patients with confirmed pCR.

Case no.	Outcome	DFS (months)	Relapse site	Adjuvant chemotherapy	Stage	Involved ALNs	Subtype	Ki-67 (%)
1	Alive	34.0	-	-	IIIB	0	Luminal^a^	14.7
2	Alive	2.9	Brain	Docetaxel, trastuzumab	IIIC	0	HER2^b^	7.1
3	Dead	7.9	Brain	Tegafur	IIIC	1	TN^c^	55.2
4	Alive	8.4	-	-	IIIA	1	TN^c^	42.0

a Luminal type;

b human epidermal growth factor receptor 2 (HER2)-positive type;

c triple-negative type.

pCR, pathological complete response; DFS, disease-free survival; ALNs, axillary lymph nodes.

**Table V.** Characteristics of the NAC and AT groups.

Characteristic	NAC group (n=22)	AT group (n=31)	P-value
Age [years (range)]	55 (33–72)	52 (37–77)	0.564
Histological type			0.904
Invasive ductal carcinoma	19	27	
Invasive lobular carcinoma	1	2	
Mucinous carcinoma	2		
Metaplastic carcinoma		2	
Intrinsic subtype			0.409
Luminal type	6	14	
HER2-positive type	9	9	
Triple-negative type	7	8	
Initial stage			0.040
Stage IIIA	10	23	
Stage IIIB	7	7	
Stage IIIC	5	1	
Events of recurrence			0.693
Local recurrence	2	6	
Distant metastasis	12	21	

NAC, neoadjuvant chemotherapy; AT, adjuvant therapy; HER2, human epidermal growth factor receptor 2.
